# Association of depression with gastroesophageal reflux disease, and the mediating role of risk factors: a Mendelian randomization study

**DOI:** 10.3389/fpsyt.2024.1425730

**Published:** 2024-11-04

**Authors:** Hui Duan, Lan Tao, Kaiwen Wu, Qian Li, Xinxu Zhou, Peiwen Dong, Xiaobin Sun, Lin Lin, Xiaolin Ma, Rong Zhao, Qiong Wang

**Affiliations:** The Third People’s Hospital of Chengdu, The Affiliated Hospital of Southwest Jiaotong University, Chengdu, Sichuan, China

**Keywords:** depression, gastroesophageal reflux, educational status, Mendelian randomization analysis, GERD

## Abstract

**Background:**

Growing evidence suggests that depression affects gastroesophageal reflux disease (GERD). But, the relationship between depression and GERD is unclear. To examine the relationship between depression and the risk of GERD, as well as the mediating role of risk factors.

**Methods:**

We found genetic variants associated with GERD (N = 78,707) and depression (N = 500,199 (excluding 23 and Me) from the largest genome-wide association study and we applied two-sample Mendelian randomization (MR) to find out if they are related. We further used two-step MR to find the mediating factors.

**Results:**

The results found a causal link between depression and GERD, inverse-variance weighted (IVW), risk OR 2.149 (95% CI, 1.910 to 2.418; *P* <0.001). F-statistics for all instrumental variables (IVs) were greater than 10. Multivariate MR maintained the significance of the depression-GERD link even after adjusting for body mass index (BMI), waist-to-hip ratio (WHR), and educational attainment (EA). Mediation analysis revealed that increased depression is associated with lower EA (OR = 0.94; 95% CI, 0.89 to 0.99; P = 0.03), while EA itself significantly impacts GERD risk (OR = 0.25; 95% CI, 0.18 to 0.34; P = 8.24 × 10^-9^). Ultimately, EA mediates the effect of depression on GERD (OR = 1.09; 95% CI, 1.01 to 1.18; P = 0.04), accounting for 11.4% of the mediated effect.

**Conclusions:**

Depression is associated with an increased risk of developing GERD, with some of the effects mediated by EA. This result may provide important information for the prevention and intervention of depression and GERD.

## 1 Introduction

GERD is a condition in which the contents of the stomach and duodenum reflux into the esophagus, causing symptoms such as acid reflux and heartburn (1, 2). Reflux can also cause tissue damage in the mouth, throat, airway, and other tissues near the esophageal collar, resulting in extra-esophageal manifestations such as cough, hoarseness, pharyngitis, asthma, idiopathic pulmonary fibrosis, etc (3). GERD is one of the most common chronic diseases in the world. It is becoming increasingly prevalent, especially in some developing countries (4, 5). GERD is a serious threat to people’s quality of life and physical and mental health, placing a huge burden on patients and their families (6–8). Nowadays, GERD is recognized as an important health problem in the world.

Many studies have researched the relationship between psychological factors and gastrointestinal disorders (9, 10). The brain influences gastrointestinal functions, such as stress can affect the gastrointestinal tract, leading to gastrointestinal symptoms and diseases. Psychological factors influence functional gastrointestinal disorders, such as gastroesophageal reflux disease, through the gut-brain axis. Additionally, changes in psychological factors can lead to the development of gastrointestinal disorders or symptoms, and there have been a number of GERD studies that have shown the effects of psychological factors, especially depression and anxiety, on GERD. When anxiety or depression occurs, treatment of functional disorders becomes difficult and leads to adverse outcomes (11). Currently, a number of reflux studies have shown that depression has an effect on reflux patients; however, there is some inconsistency in the results of these studies (1, 11–15). Some studies have analyzed the effect of psychological factors on different types of GERD (1, 11–15). Some studies have not found a significant relationship between depression and GERD (1, 14–16).

Traditional epidemiological studies, such as randomized controlled trials, are often costly in terms of human, material and financial resources. Moreover, the interventions given in randomized controlled trials may be inhumane. Mendelian randomization is a more scientific and convenient alternative to using SNPs as an instrumental variable to estimate the effects of exposure factors on outcomes (16). Problems such as reverse causation and confusion are minimized by the fact that genetic variants are present prior to the onset of the disease (17). The relationship between gastroesophageal reflux and depression has been studied by two-way Mendelian mediators (Korean subjects) (18). However, there are no relevant analyses for European populations, and there is no relevant study on whether there is a mediating relationship between depression and GERD.

The data used in the analysis were extracted from the pooled statistics of the world’s largest database of genetic association studies (GWASs) to research the relationship between depression and GERD and its mediators. Considering the effect of depression on obesity and education, we used a two-step MR analysis to find the mediating pathway of depression to GERD through obesity and education-related phenotypes.

## 2 Methods

### 2.1 Mendelian randomization hypothesis

MR studies have utilized SNPs, which are closely related to exposure, as instrumental variables (IVs) to find the effect of exposure on outcomes. Risk estimates for the association between depression and GERD were then derived from inverse variance weighted (IVW) principal analyses as well as sensitivity analyses by weighted median and MR-Egger regression.

By analyzing the instrumental variables of the pooled data, the SNPs, which is strongly correlated with exposure, were found as an instrumental variable. The following three assumptions then need to be met to make the MR findings valid (19, 20): (1) Genetic variation is strongly associated with exposure; (2) Genetic variation is independent of any confounders associated with exposure-outcome; (3) These variants do not independently influence the results.

**Figure 1:**
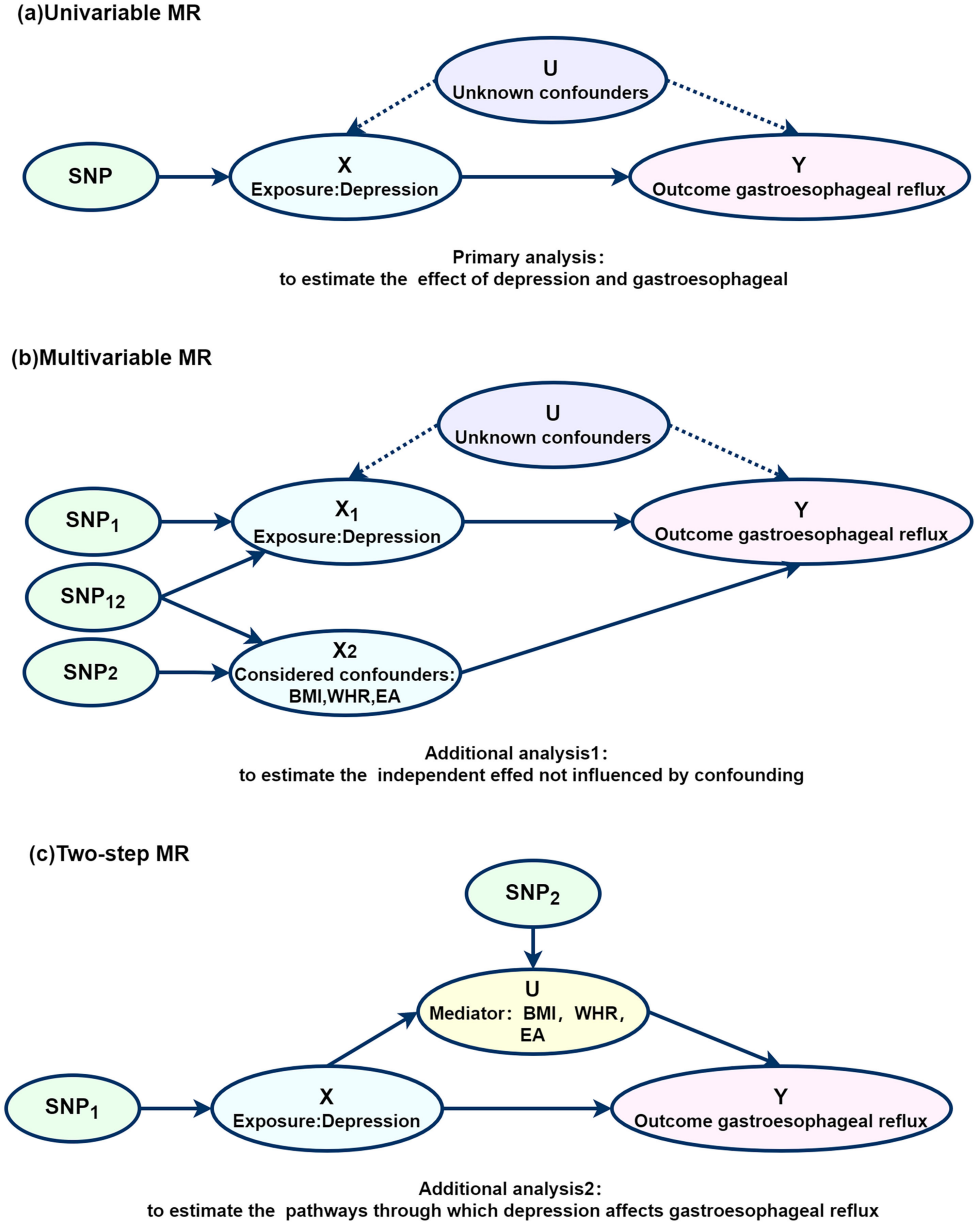
Diagram of the Mendelian randomization and mediation analysis. **(A)** Univariable MR; **(B)** Multivariable MR; **(C)** Two-step MR. MR, Mendelian randomization; SNP, Single nucleotide polymorphisms.

First, we performed two-sample MR analysis to assess the relationship between depression and GERD. Then multivariate MR was utilized to further assess the direct effect of depression on GERD, independent of other factors. Finally, we applied two-step MR analysis to analyze and assess whether an intermediate factor such as EA mediates the relationship between depression and GERD (**Figure 1**).

### 2.2 Data sources and selection of tools

The characteristics and details of the datasets included in this study are shown in **Table 1**. The data for this study were obtained from the publicly available GWAS database; ethical approval was obtained for the original studies for which data were used in this article. The summary statistics for depression were obtained from the GWAS study of self-reported clinical diagnosis of depression in European populations published by Howard et al. We included studies comprising 500,199 depressed patients and 329,443 controls (excluding the 23 and Me cohort). Inclusion included UK Biobank (127552 patients, 233763 controls) (21) and Psychiatric Genomics Consortium (43204 patients, 95680 controls) (22). Because 23 and me GWAS study was based on a web page questionnaire, it was excluded (23). GERD refers to a chronic digestive disorder where stomach acid or bile irritates the esophagus lining. Symptoms include heartburn, regurgitation, and discomfort (24). Genetic variables and summary statistics for GERD from the GWAS study of GERD in European populations including 78,707 GERD patients and 288,734 controls published by Ong, J.S. et al. (25–28). Genetic variables and summary data for BMI and WHR were obtained from the Society for the Genetic Investigation of Human Traits (GIANT) incorporating 694,648 samples. Educational attainment (EA) refers to the highest level or degree of education an individual has completed, typically used to describe a person’s educational level and qualifications (29). EA data were from the Gene Discovery and Multigene Prediction in GWAS study of the educational attainment of 1.1 million people, including 1,100,000 samples. The included GWAS studies were all based on European population.

**Table 1:** Details on the characteristics of each included dataset.

Phenotype	Data source	Total sample size	Reference genome	Imputation panel	Population	SNPs
Depression	Howard, D.M., et al. Genome-wide meta-analysis of depression identifies 102 independent variants and highlights the importance of the prefrontal brain regions. Nat Neurosci 22, 343–352(2019).	500,199 (excluding 23andMe)	GRCh37	1000G Phase 3/UK10K/HRC panel	European	8.1M
gastroesophageal reflux	Ong, J. S. et al. Multitrait genetic association analysis identifies 50 new risk loci for gastroesophageal reflux, seven new loci for Barrett’s oesophagus and provides insights into clinical heterogeneity in reflux diagnosis. Gut. 71, 1053-1061(2022).	78 707 cases, 288 734 controls	GRCh37	1000G Phase 3	European	10.1M
BMI	Pulit SL et al. GIANT Consortium. Meta-analysis of genome-wide association studies for body fat distribution in 694 649 individuals of European ancestry. Hum Mol Genet 28, 166–74(2019).	694, 648	GRCh37	HRC panel	European	27.4M
WHR	Pulit SL, Stoneman C, Morris AP et al. GIANT Consortium. Meta-analysis of genome-wide association studies for body fat distribution in 694 649 individuals of European ancestry. Hum Mol Genet 28, 166–74(2019).	694, 648	GRCh37	HRC panel	European	27.5M
EA	Lee JJ, Wedow R, Okbay A, et al. Gene discovery and polygenic prediction from a genome-wide association study of educational attainment in 1.1 million individuals. Nat Genet 50, 1112-1121(2018).	1,100,000	GRCh37	1000G Phase 3	European	10M

SNP, single nucleotide polymorphisms;

1000 G, the 1000 Genomes Project; HRC, the Haplotype Reference Consortium.

### 2.3 Testing instrument strength and statistical power

The F-statistic, as a measure of instrument strength, is calculated based on the interplay of genetic variants (R2), sample size (N), and the number of instruments (k) (30). The R^2^-specific calculation formula is as follows: R^2^ = 2 × minor allele frequency (MAF) × (1-MAF) × beta.exposure^2^, where R^2^ is the proportion of variance explained in the instrumental variable. An F-statistic greater than or equal to 10 indicates a relatively low risk of bias. To gauge the robustness of our study, we employed the methodology outlined by Burgess (30). In essence, this approach calculates statistical power by considering variables such as the sample size in genome-wide association studies (GWAS), the proportion of cases in case-control GWAS, and the variance explained by the genetic tools related to the exposure (31, 32).


$$\text{F}=\frac{N-k-1}{k}\times \frac{R^{2}}{1-R^{2}}$$

### 2.4 Two-sample Mendelian randomization

Two-sample Mendelian randomization was employed for each exposure, and the primary MR analysis utilized the IVW method (33, 34). This approach amalgamated the Wald ratio estimates of each SNP into a singular causal estimate for each risk factor, calculated by dividing SNP-outcome association by SNP-exposure association (33). The results were presented as odds ratios (ORs). The more symptomatic a depressed patient is, the higher the risk of GERD.

To address potential biases introduced by pleiotropic instrumental variables, sensitivity analyses were conducted. The potential presence of horizontal pleiotropy was assessed using MR-Egger, where deviation from zero (*P* < 0.05) indicated horizontal pleiotropic bias (35, 36). The slope coefficient from the MR-Egger regression provides a consistent estimate of the causal effect in the presence of horizontal pleiotropy.

Additionally, we used the weighted median method for the sensitivity analysis. The weighted median method estimate causal effects from the median of weighted empirical density functions of individual SNP effect estimates, allowing up to 50% of variant information to violate the MR assumption in the presence of horizontal pleiotropy (37).

Multiplicity was assessed using MR-PRESSO by comparing the observed distances of all variables to the regression line with the expected distances under the null hypothesis of no multiplicity (38). To evaluate the influence of individual variants on the observed associations, a leave-one-out analysis was performed.

### 2.5 Multivariable MR

Considering that depression is interrelated with several factors, we performed multivariate MR (39) for simultaneous estimation of the direct effect of depression on GERD depending on other influences (BMI, WHR and EA).

### 2.6 Mediation analysis

A two-step MR analysis was used to assess mediation effects for some of the significant correlations. First, assessing the causal effects of depression on potential mediators. After that, to estimate the effect of potential mediators on GERD risk, genetic tools strongly associated with potential mediators were used.

Where depression was found to influence the mediator, and the mediator in turn influenced GERD, we used a ‘product of coefficients’ approach to estimate the proportion of mediated effects from depression to GERD.

## 3 Results

### 3.1 Two-sample Mendelian randomization

For the selection of genetic instrumental variables, using genetic variants associated with GERD and depression from the most recent European pedigree GWAS, we performed two-sample MR (**Table 1**). The SNPs were screened for two-sample Mendelian analysis, which showed a causal relationship between depression and GERD.

Depressive disorder was related to a higher risk of GERD, inverse-variance weighted IVW, OR 2.149 (95% CI, 1.910 to 2.418; *P* <0.001). In addition, MR- Egger, weighted median, and MR-PRESSO methods gave consistent results, details are provided in **Table 2**. F-statistics for all IVs were greater than 10 (**Supplementary Table S1**).

**Table 2:** Causal effects of depression on gastroesophageal reflux.

Exposure	Method	nSNP	OR (95%CI)	P-value
Depression	Inverse variance weighted	43	2.149 (1.910-2.418)	<0.001
	MR Egger	43	1.863 (1.000-3.472)	0.060
	Weighted median	43	1.989 (1.779-2.223)	<0.001
	MR-PRESSO (4outlier-corrected)	39	2.154 (1.964-2.362)	<0.001
	excluding palindromic SNPs	39	2.182 (1.920-2.479)	<0.001
	excluding pleiotropic SNPs	28	2.060 (1.781-2.383)	<0.001

**Figure 2:**
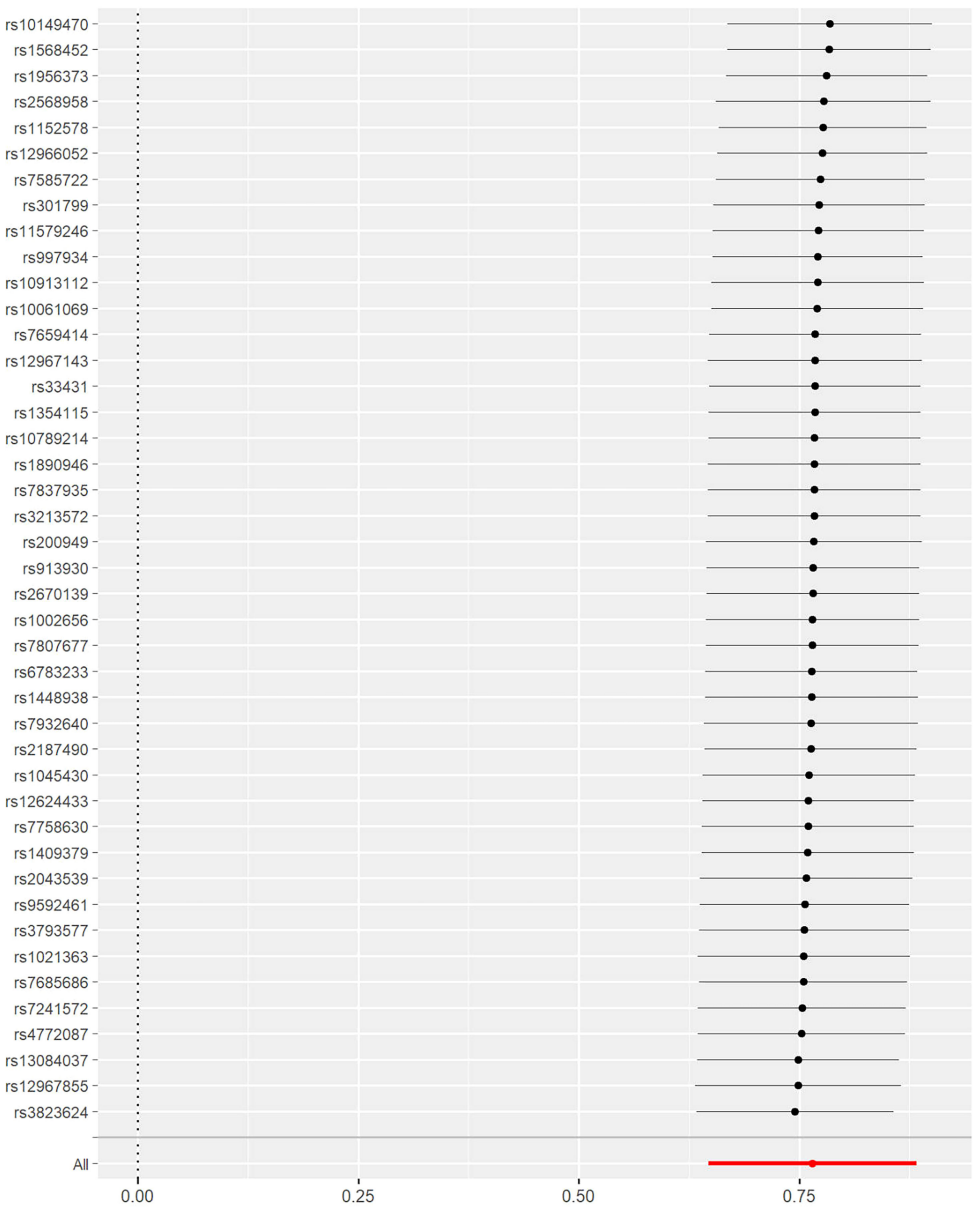
Results from the leave-one-out analysis which excluded each single nucleotide polymorphism (SNP) to estimate the effects of depression-related traits on gastroesophageal reflux.

Furthermore, IVW was used to leave-one-out analysis, excluding each SNP to estimate the effect of depression-related traits on GERD. The results showed that no single SNP drove this outcome, suggesting an overall combinatorial pattern with depression and GERD (**Figure 2**).

### 3.2 Multivariable MR

**Figure 3:**
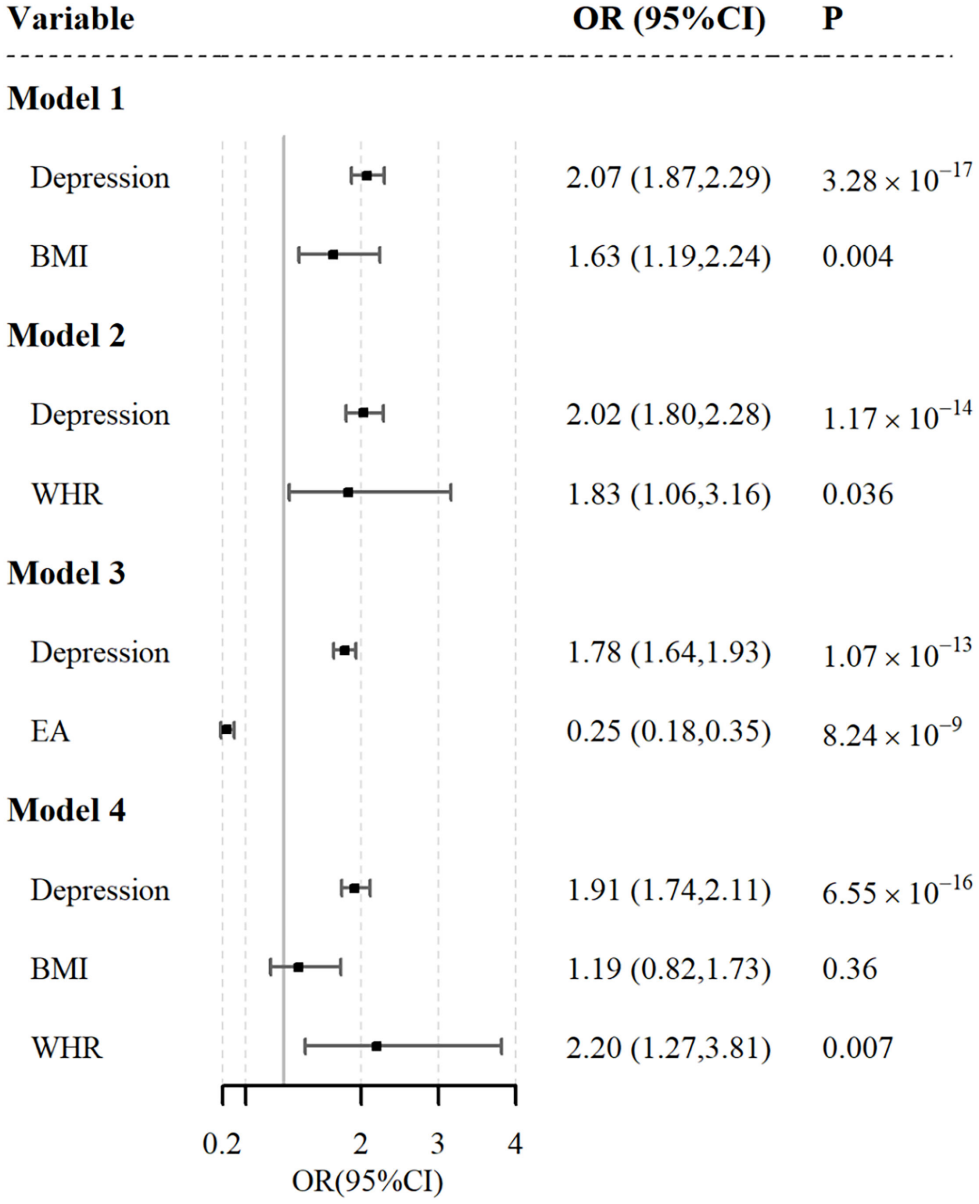
Independent effect of depression on the risk of gastroesophageal reflux using multivariable Mendelian randomization analysis. Model 1: independent effect of depression on gastroesophageal reflux after adjusting BMI; Model 2: independent effect of depression on gastroesophageal reflux after adjusting WHR; Model 3: independent effect of depression on gastroesophageal reflux after adjusting EA; Model 4: independent effect of depression on gastroesophageal reflux after adjusting BMI & WHR.

We applied multivariate MR to estimate the independent effect of depression on GERD under other conditions (**Figure 3**). As shown, depression and GERD remained statistically significant after correction for BMI(OR1.63; 95% CI, 1.190 to 2.400; P =0.004), WHR(OR1.63; 95% CI, 1.190 to 2.400; P =0.004), and EA (OR 0.25; 95% CI, 0.180 to 0.350; P =8.24×10-19) (*P*<0.05).

### 3.3 Mediation analysis

**Figure 4:**
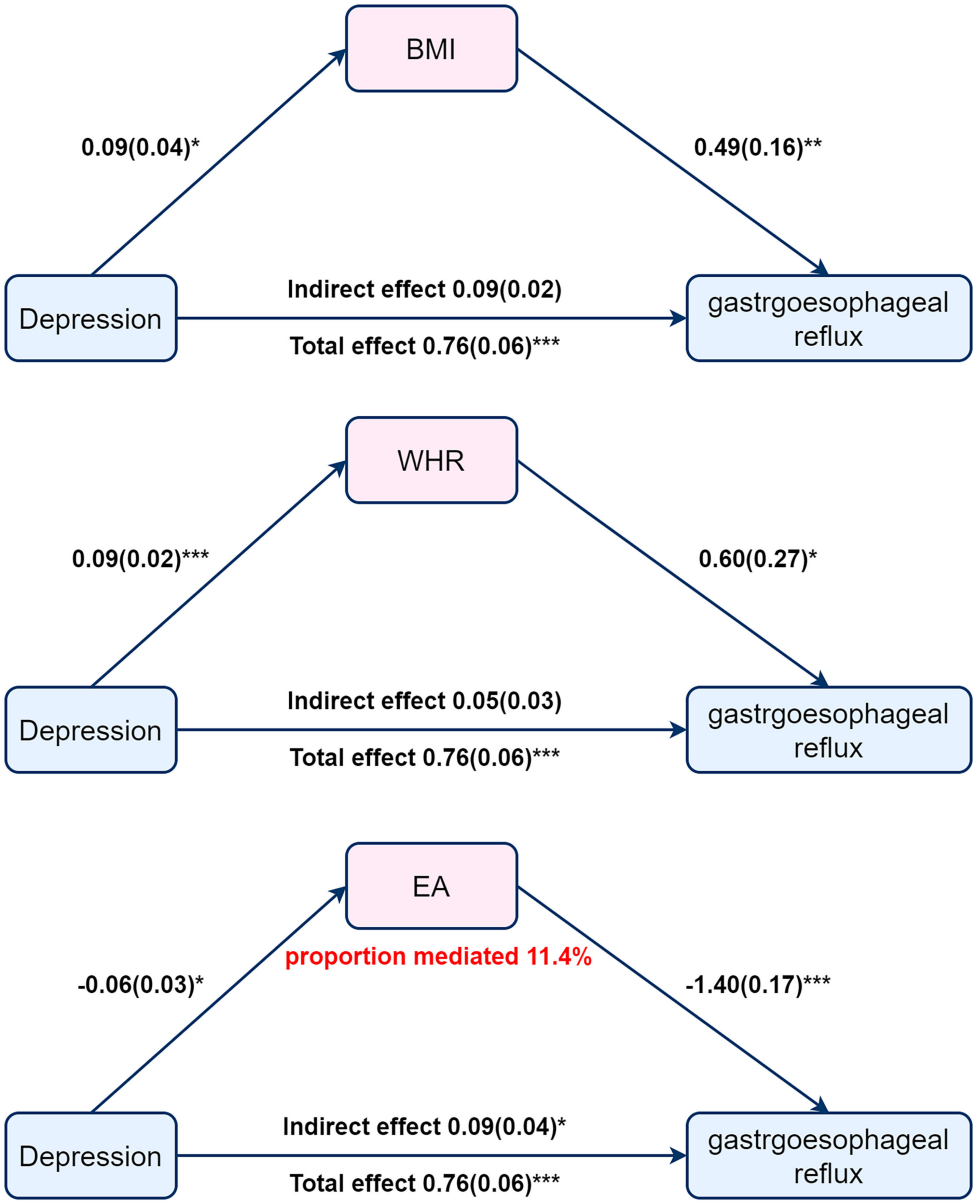
Mediation Mendelian Randomization between depression and gastroesophageal reflux adjusted for BMI, WHR or EA. Beta (SE). *P* value * <0.05, **<0.01, ***<0.001.

We used a two-step MR analysis to examine the mediating pathway from depression to GERD through obesity and EA-related phenotypes such as body mass index (BMI), waist-to-hip ratio (WHR), and educational attainment (EA) (**Figure 4**).

Initial step, genetic instruments for depression were used to estimate the effects of exposure on BMI, WHR, and EA. Of these three mediators, we found a causal relationship only between depression and EA, with increased depression associated with lower EA (OR =0.94; 95% CI, 0.89 to 0.99; *P* = 0.03) (**Figure 4**). And then, we assessed the causal effects of BMI, WHR, and AE on GERD risk. We found evidence that EA influences GERD (OR =0.25; 95%CI, 0.18 to 0.34; *P*=8.24×10^−9^) (**Figure 4**). Finally, we find a mediating role for EA in the effect of depression on GERD (OR=1.09; 95% CI,1.01 to 1.18; P=0.04) with a mediated proportion of 11.4% (**Figure 4**). The results suggest that educational attainment mediates the effect of depression in the development of GERD. Increased depression is often accompanied by lower EA, and low EA leads to higher GERD incidence.

## 4 Discussion

Several key risk factors contribute to the pathogenesis of gastroesophageal reflux disease (GERD), including psychological factors such as anxiety and depression, as well as sociodemographic and lifestyle factors like lower educational attainment, obesity, and dietary habits (40–45). Previous clinical studies have demonstrated significant associations between educational attainment (EA), body mass index (BMI), waist-to-hip ratio (WHR), and the occurrence of GERD. To explore these relationships, we examined body mass index (BMI), waist-to-hip ratio (WHR), educational attainment (EA), and depression. Using genetic variants as instrumental variables, we estimated the effects of these factors on GERD through Mendelian randomization (MR) analysis. Our results demonstrated a causal association between depression and increased GERD risk. Importantly, the findings were consistent across multiple MR methods, suggesting minimal risk of bias due to horizontal pleiotropy. Further mediation analysis indicated that the influence of depression on GERD risk is partially mediated by EA.

The brain-gut axis (GBA) elucidates the interplay between gastrointestinal diseases, gut microbiota, and neurological symptoms, revealing the relationship between depression and gastroesophageal reflux disease (GERD). In particular, the abnormal secretion of brain-gut peptides may reduce the pressure of the lower esophageal sphincter, which not only promotes the development of GERD but may also impact the onset of depression. Therefore, these findings highlight the necessity for further in-depth research on these biological pathways to better understand these complex diseases and to develop more effective intervention measures (46–50). Serotonin (5-HT), a key neurotransmitter in the gut nervous system, regulates intestinal sensation and motility. Its overactivation in GERD disrupts gastrointestinal motility while also affecting depression (51). Both GERD and depression show significant gut microbiota abnormalities that are crucial for their development and progression through the microbiota-gut-brain axis. Dysregulation of the hypothalamic-pituitary-adrenal (HPA) axis also plays a role in both conditions, suggesting that restoring HPA regulation could alleviate their co-occurrence (52). Additionally, immune system changes in pro-inflammatory and anti-inflammatory cytokines contribute to GERD and depression (53). Thus, depression and GERD interact through multiple physiological and behavioral pathways (53). Future research should focus on interventions targeting these pathways to improve management strategies for both conditions.

EA may mediate the relationship between depression and gastroesophageal reflux disease (GERD) through the brain-gut axis hypothesis. Research indicates that higher EA is beneficial for preventing and treating GERD (53). Educated individuals typically enjoy better economic and social status, stable jobs, healthier lifestyles, social support, and improved access to healthcare (53–60). Conversely, those with lower education levels often experience higher stress (61), which negatively affects gut microbiota composition, particularly reducing beneficial populations like Lactobacillus and Bifidobacterium (62). This decline in probiotics can increase GERD incidence (63). Additionally, lower-educated individuals are more likely to face occupational hazards that disrupt gastrointestinal flora, while environmental contaminants from human activities exacerbate these issues (63–66). Their diets tend to be less nutritious, further compromising gut health and elevating GERD risk, higher EA acts as a protective factor for GERD, underscoring the importance of health education on lifestyle and dietary management in GERD treatment (67, 68). Studies show that greater EA enhances disease coping abilities, reduces symptoms, and lowers healthcare costs (69–72). Future research should focus on targeted interventions to improve EA and its potential protective effects against GERD.

Studies indicate a strong correlation between depression and educational attainment (EA), explained by distribution and socialization mechanisms. Individuals with higher education generally possess greater economic and social resources, enhancing their resilience to depression (73–76). Higher education is linked to increased income, better social status, and a lower likelihood of issues such as unemployment and divorce, contributing to reduced depression rates. Socialization also plays a key role; education fosters stronger problem-solving skills and coping strategies, enabling individuals to navigate life’s challenges more effectively. Those with higher education levels are more likely to cultivate robust social networks for support, further lowering their depression risk (77–80). These mechanisms elucidate the relationship between depression and EA, aligning with our Mendelian analysis findings. Future research should explore interventions that leverage these mechanisms to mitigate depression among lower-educated populations.

We conducted a mediation analysis using two-step MR and found that a small proportion of the effect of depression on GERD was mediated through EA. In the initial step, univariate MR identified a causal relationship between depression and EA, with depression negatively correlated with EA. It has been reported that depression is negatively correlated with educational attainment, and that low educational attainment and depression interact in a vicious cycle (81). This is consistent with the results estimated in our first step of the analysis. The second step of magnetic resonance provides evidence of genetically determined correlations between high EA and low GERD incidence odds. Some studies have reported evidence of causal risk factors for EA and GERD or related phenotypes (53). This is consistent with the conclusions reached in the second step of our mediation analysis. Furthermore, as we expected, there was no significant causal relationship between body fat distribution (as assessed by WHR and BMI), and GERD risk. Hence, the effect of EA on GERD has been well documented in these studies. The relationship between education attainment (EA), depression, and GERD has garnered significant attention among researchers. The prevailing view suggests that EA may mediate the connection between gastroesophageal reflux and depression through several mechanisms. Firstly, EA can shape individuals’ understanding and perception of health issues, including symptoms of gastroesophageal reflux. This cognitive influence may affect how individuals interpret and manage their symptoms, potentially influencing their susceptibility to depression (44). Secondly, EA might prompt behavioral changes such as dietary adjustments or stress management, which can alleviate gastroesophageal reflux symptoms and indirectly reduce the risk of depression (82). Additionally, enhanced EA could bolster coping skills and social support systems, recognized as protective factors against depression. This may alleviate the psychological burden associated with gastroesophageal reflux, thus lowering the likelihood of depression (83). These pathways suggest that EA, by influencing cognitive, behavioral, and psychosocial factors, may play a significant mediating role in the relationship between gastroesophageal reflux and depression.

Our findings suggest an association between GERD, depression, and educational attainment (EA), the study’s design does not allow for definitive conclusions regarding causality. We collected genetic information from the world’s largest database of GWAS, allowing us to include as many exposure tools as possible, thus improving statistical power. European ancestry dominated all datasets in this study; therefore, there is no potential bias in the classification of ethnicity. Undeniably, the relevant results of this study should be interpreted in the context of MR. Despite the selection of strongly correlated single-nucleotide polymorphisms, genetic variation can only explain a portion of the total variation in depression and cannot be considered representative of all exposures. We cannot completely rule out violations of the independence assumption because we do not know the biological role of the genetic tools, and exclusionary restrictions, especially with respect to pleiotropy (84). However, we used a variety of methods to infer the robustness of the results, including sensitivities analyzed using MR-PRESSO, weighted median, and MR-Egger. Types of depression, such as atypical depression and melancholic depression, were not broken down in the depression GWAS we used. Finally, estimates from MR studies may also be affected by environmental and social factors. This bias can be avoided by using in-home GWASs in the future (85).

## 5 Conclusion

We found genetic evidence that depression is associated with GERD. EA, as a mediator, mediated this effect to a lesser extent. Reducing depression has a protective effect on the risk of GERD, and low depression is often associated with high EA, higher EA helps prevent and treat GERD. The potential impact of our findings on GERD prevention warrants validation in randomized clinical trials.

## Data Availability

The datasets presented in this study can be found in online repositories. The names of the repository/repositories and accession number(s) can be found in the article/**Supplementary Material**.
