# Supplementary material for: Association of depression with gastroesophageal reflux disease, and the mediating role of risk factors: a Mendelian randomization study
Source: Front Psychiatry. 2024 Nov 4;15:1425730. doi: 10.3389/fpsyt.2024.1425730 (PMC11600978; doi:10.3389/fpsyt.2024.1425730)
Supplement: Supplementary file 1 [file DataSheet1.pdf]

Table S1 Characteristics of GWAS-identified depression-associated genetic instruments.

SNP	Chromosome	Position	Effect allele	Other allele	Effect allele frequency	Beta	Standard Error	*P*-value	*F*-statistic
rs1002656	1	37192741	T	C	0.7033	-0.0266	0.0038	3.74E-12	147.7
rs10789214	1	67146817	T	C	0.5661	0.0193	0.0035	4.44E-08	91.5
rs10890020	1	73668836	A	G	0.5156	-0.0277	0.0035	4.03E-15	191.8
rs10913112	1	175913828	T	C	0.3767	-0.0264	0.0036	3.40E-13	163.8
rs113188507	1	80809636	A	G	0.2838	0.0221	0.0039	1.87E-08	99.3
rs11579246	1	50559162	A	G	0.9067	0.0381	0.0061	5.71E-10	122.9
rs1466887	1	37709328	T	C	0.5511	-0.0199	0.0036	4.12E-08	98.0
rs169235	1	181740924	A	G	0.7530	-0.0229	0.0041	2.98E-08	97.6
rs17641524	1	197704717	T	C	0.2091	-0.0320	0.0043	1.52E-13	169.5
rs1890946	1	52342427	T	C	0.4671	-0.0235	0.0035	2.68E-11	137.6
rs2568958	1	72765116	A	G	0.6156	0.0373	0.0036	8.47E-25	329.6
rs301799	1	8489302	T	C	0.5694	-0.0250	0.0035	1.36E-12	153.3
rs72710803	1	177428018	A	C	0.9121	-0.0410	0.0062	5.29E-11	134.9
rs12052908	2	22503044	A	T	0.5325	-0.0220	0.0035	4.44E-10	120.6
rs1226412	2	157111313	T	C	0.7917	0.0256	0.0043	3.46E-09	108.1
rs1568452	2	58012833	T	C	0.3851	0.0248	0.0036	8.12E-12	145.7
rs62188629	2	208044470	A	G	0.3136	0.0236	0.0038	7.13E-10	120.0
rs7585722	2	8681912	T	C	0.8458	-0.02	0.0048	2.68E-08	94.4

		8				69			
rs1095626	3	157977962	T	C	0.5799	-0.0264	0.0035	7.13E-14	169.9
rs13084037	3	49214066	A	G	0.7740	-0.0245	0.0042	7.08E-09	105.1
rs141954845	3	61192911	A	G	0.3880	0.0229	0.0037	8.15E-10	124.6
rs4346585	3	44736493	T	C	0.6960	-0.0236	0.0038	7.13E-10	117.9
rs6783233	3	117509984	T	C	0.2833	0.0218	0.0039	2.90E-08	96.5
rs7624336	3	53244151	T	G	0.2087	0.0238	0.0043	3.96E-08	93.6
rs34937911	4	42110353	T	C	0.8838	0.0304	0.0055	4.13E-08	95.0
rs35553410	4	131237381	T	C	0.7462	-0.0244	0.0040	1.42E-09	112.8
rs45510091	4	123186393	A	G	0.9472	0.0448	0.0080	1.83E-08	100.4
rs7659414	4	177350956	A	C	0.5782	-0.0201	0.0035	1.20E-08	98.6
rs7685686	4	3207142	A	G	0.5753	0.0202	0.0036	2.57E-08	99.8
rs10061069	5	93071630	C	G	0.2212	-0.0275	0.0042	8.15E-11	130.4
rs11135349	5	164523472	A	C	0.4713	-0.0295	0.0035	6.04E-17	217.0
rs30266	5	103972357	A	G	0.3296	0.0308	0.0037	1.45E-16	209.8
rs3099439	5	87545318	T	C	0.5288	-0.0276	0.0035	5.05E-15	190.0
rs60157091	5	61509655	T	C	0.5150	0.0200	0.0035	1.42E-08	100.0
rs1933802	6	105365891	C	G	0.4536	-0.0223	0.0035	2.57E-10	123.3
rs200949	6	27835435	A	G	0.8744	0.0480	0.0053	2.53E-19	253.3
rs2029865	6	165121844	A	T	0.4534	-0.0201	0.0035	1.20E-08	100.2
rs2876520	6	142996618	C	G	0.5271	-0.0230	0.0036	2.29E-10	131.9
rs725616	6	147950422	T	C	0.3644	0.0204	0.0036	1.87E-08	96.4
rs7758630	6	1013873	A	T	0.4051	-0.02	0.0036	5.56E-10	122.1

		04				25			
rs9363467	6	66565703	T	C	0.6035	0.0237	0.0036	6.44E-11	134.5
rs16887442	7	82936909	T	C	0.4347	0.0203	0.0035	8.62E-09	101.3
rs2043539	7	12253880	A	G	0.4177	0.0273	0.0035	9.89E-15	181.4
rs2247523	7	82454404	C	G	0.5319	-0.0207	0.0035	4.38E-09	106.8
rs3823624	7	2110346	T	C	0.8067	0.0272	0.0045	1.99E-09	115.4
rs58104186	7	109099919	A	G	0.4689	0.0237	0.0035	1.82E-11	140.0
rs7807677	7	117502574	T	C	0.5505	0.0237	0.0035	1.82E-11	139.1
rs67436663	8	71347626	C	G	0.2402	-0.0259	0.0042	9.37E-10	122.5
rs7837935	8	65562019	T	G	0.1522	-0.0292	0.0049	3.34E-09	110.1
rs10817969	9	119731045	T	G	0.7173	0.0261	0.0039	3.11E-11	138.2
rs1354115	9	2983774	A	C	0.6243	0.0210	0.0036	7.08E-09	103.5
rs1982277	9	11513019	T	C	0.7594	0.0279	0.0041	1.45E-11	142.3
rs263645	9	17016503	A	T	0.5438	0.0221	0.0035	3.70E-10	121.2
rs2670139	9	126634255	T	C	0.7609	-0.0266	0.0041	1.21E-10	128.8
rs34653192	9	31124452	C	G	0.3196	-0.0229	0.0038	2.23E-09	114.1
rs3793577	9	23737627	A	G	0.4665	-0.0229	0.0035	8.41E-11	130.6
rs59283172	9	25232978	A	G	0.1069	-0.0329	0.0057	1.02E-08	103.4
rs7030813	9	36999369	T	C	0.3736	0.0253	0.0036	3.07E-12	149.9
rs913930	9	120484009	A	G	0.6433	-0.0208	0.0037	2.42E-08	99.3
rs1021363	10	106610839	A	G	0.3547	0.0303	0.0037	4.41E-16	210.3
rs997934	10	1795194	T	C	0.3795	0.0198	0.0036	4.81E-08	92.4
rs1448938	11	3089282	A	G	0.4171	0.02	0.0035	1.30E-09	111.4

		4				14			
rs198457	11	61471678	T	C	0.1925	-0.0292	0.0046	2.99E-10	132.6
rs2187490	11	118713180	T	G	0.9106	-0.0338	0.0061	3.82E-08	93.1
rs2509805	11	57650796	T	C	0.3209	0.0220	0.0038	9.17E-09	105.5
rs57344483	11	127022560	A	G	0.9259	-0.0380	0.0068	1.82E-08	99.1
rs58621819	11	65314830	A	T	0.7903	-0.0245	0.0043	1.57E-08	99.5
rs61902811	11	113370758	A	G	0.3682	-0.0257	0.0036	1.40E-12	153.8
rs7117514	11	70544937	A	G	0.5417	-0.0204	0.0035	7.29E-09	103.4
rs7932640	11	88744425	T	C	0.4417	0.0281	0.0035	1.62E-15	194.9
rs10774600	12	110741356	T	C	0.1656	-0.0267	0.0048	3.39E-08	98.6
rs3213572	12	121205078	A	G	0.4745	0.0217	0.0035	7.61E-10	117.5
rs56314503	12	84465022	T	G	0.7487	-0.0254	0.0040	2.95E-10	121.5
rs78337797	12	23987925	T	G	0.8781	0.0306	0.0055	3.37E-08	100.3
rs1343605	13	53647048	A	C	0.3840	0.0313	0.0036	6.23E-18	231.9
rs1409379	13	31907741	T	C	0.7641	0.0249	0.0041	1.67E-09	111.8
rs4772087	13	99115041	T	C	0.3732	0.0227	0.0036	3.91E-10	120.6
rs9545360	13	80826373	A	C	0.1807	-0.0271	0.0046	5.02E-09	108.8
rs9592461	13	66941792	A	G	0.4874	0.0216	0.0035	9.10E-10	116.6
rs10149470	14	104017953	A	G	0.4869	-0.0267	0.0035	3.72E-14	178.2
rs1045430	14	75130235	T	G	0.4792	-0.0253	0.0035	7.31E-13	159.9
rs1152578	14	64697037	T	C	0.4357	-0.0218	0.0035	6.36E-10	116.9
rs1956373	14	60141822	T	G	0.7436	-0.0226	0.0040	2.06E-08	97.4
rs6199028	14	4207472	A	G	0.5083	-0.02	0.0035	1.68E-13	169.1

8		6				60			
rs34488670	15	47684936	T	C	0.7887	-0.0252	0.0043	6.03E-09	105.9
rs8037355	15	37643831	T	C	0.5556	-0.0233	0.0035	3.94E-11	134.1
rs12923444	16	21639710	A	C	0.5625	-0.0214	0.0035	1.30E-09	112.8
rs56887639	16	13755530	A	G	0.7264	-0.0278	0.0039	1.51E-12	153.7
rs7193263	16	6315880	A	G	0.6679	-0.0239	0.0038	4.33E-10	126.8
rs7198928	16	7666402	T	C	0.6159	0.0239	0.0036	4.45E-11	135.2
rs7200826	16	13066833	T	C	0.2551	0.0280	0.0040	3.74E-12	149.1
rs75581564	17	27363750	A	G	0.1165	0.0301	0.0054	3.17E-08	93.3
rs12966052	18	52751639	C	G	0.1805	-0.0314	0.0046	1.25E-11	145.9
rs12967143	18	53099012	C	G	0.6984	-0.0312	0.0038	3.70E-16	205.2
rs12967855	18	35138245	A	G	0.3295	0.0265	0.0037	1.18E-12	155.3
rs62091461	18	52488672	T	C	0.2274	-0.0254	0.0042	1.95E-09	113.4
rs7227069	18	50731802	A	G	0.4326	0.0238	0.0035	1.50E-11	139.1
rs7241572	18	77580712	A	G	0.2010	0.0280	0.0044	2.70E-10	126.0
rs33431	19	30939989	T	C	0.6144	0.0198	0.0036	4.81E-08	92.9
rs12624433	20	44680853	A	G	0.2584	0.0233	0.0040	7.44E-09	104.1
rs143186028	20	39997404	T	G	0.1778	0.0277	0.0046	2.29E-09	112.2
rs5995992	22	41487218	T	C	0.7155	-0.0266	0.0039	1.30E-11	144.1

F-statistic was calculated using the following formulas: F=R^2 (n-2)⁄(1-R^2) and R^2=2×MAF×(1-MAF)×β^2, where F represents F-statistic, R^2 represents the phenotypic variance explained by a genetic instrument, N is the sample size, β is the estimated genetic association of SNP with the exposure, MAF is the minor allele frequency. SNP: single nucleotide polymorphisms.
